# Trade-offs between economic, environmental and social sustainability on farms using a latent class frontier efficiency model: Evidence for Spanish crop farms

**DOI:** 10.1371/journal.pone.0261190

**Published:** 2022-01-10

**Authors:** Amer Ait Sidhoum, K. Hervé Dakpo, Laure Latruffe

**Affiliations:** 1 Department of Agricultural Production and Resource Economics, Technische Universität München, Freising, Germany; 2 Natural Resources Institute Finland (Luke), Business Economics, Helsinki, Finland; 3 Université Paris-Saclay, INRAE, AgroParisTech, Economie Publique, Thiverval-Grignon, France; 4 Agricultural Economics and Policy Group, ETH Zürich, Zürich, Switzerland; 5 INRAE, GREThA, Université de Bordeaux, Bordeaux, France; Szechenyi Istvan University: Szechenyi Istvan Egyetem, HUNGARY

## Abstract

This article studies trade-offs of farms in terms of economic sustainability (proxied here by technical efficiency), environmental sustainability (proxied here by farmers’ commitment towards the environment) and social sustainability (proxied here by farmers’ contribution to on farm well-being and communities’ well-being). We use the latent class stochastic frontier model and create classes based on three separating variables, representing farms’ environmental sustainability and social sustainability. The application to a sample of Spanish crop farms shows that more environmentally sustainable farms are likely to have lower levels of technical efficiency. However, improvements in social concerns, both towards own farm and the larger community, may lead to improved technical efficiency levels. In general, our study provides evidence of trade-offs for farms between economic sustainability and environmental sustainability, but also between environmental sustainability and social sustainability.

## 1. Introduction

A resource-efficient, resilient and productive food system is seen as a fundamental vehicle for contributing to sustainable development [1]. Implementing such a transition involves the optimisation of a number of economic, environmental and social objectives, from increasing agricultural yields, reducing pollution to improving farmers’ well-being. These objectives, however, are not independent and they interact with one another. In certain situations, these interactions may have unintentional negative effects, while in other situations, the combination of these goals creates trade-offs and synergetic relationships. Therefore, the transition towards an agricultural system that balances different sustainability objectives requires identifying the goals, assessing these interactions, and designing and implementing effective management practices within heterogeneous farming environments [2].

The objective of this paper is to improve our understanding of the synergies and trade-offs between sustainability dimensions, as well as the conditions leading to an optimal management of the agricultural sector and the rural community. Our analysis focuses on the Spanish arable farms. Just as in whole Europe, the agricultural sector has undergone rapid structural change in Spain in the past decades. While the number of farms amounted to around 1 593 600 in 1970, it decreased to 945 020 in 2016. This development was mainly driven by differences in working conditions and incomes between economic sectors, as well as technological progress and efficiency improvements. Especially the use of agrochemicals and innovative machinery resulted in increased food production and lower producer prices. Intensive agricultural systems in Spain and Europe, however, created negative externalities in the form of environmental pressure and social imbalance. Against this background, investigating sustainability trade-offs in Spanish agriculture is critically important for at least three reasons. First, since farmers usually own and work on their farms (family farms are the most common type of farm in the European Union (EU))), the common expectation that market competition would mean that only the most technically efficient farmers are likely to survive and remain competitive in their industry is unlikely to hold true, and the process of adjustment will have both social and environmental consequences. Second, the challenges of sustainable agriculture are strongly connected to the new Common Agricultural Policy (CAP) post -2020. The new reform focuses on several objectives, linked to common EU goals for social, environmental, and economic sustainability in agriculture. Third, the Spanish region of Catalonia is a representative zone within many European regions with respect to production structure, agri-ecological factors and policy context.

A key difficulty in evaluating trade-offs is identifying appropriate indicators that can be used to quantitatively assess the economic, environmental and social sustainability. The economic sustainability of a farming system is based on ensuring viable economic activities in the long term. The most common indicators used in economic sustainability assessments in the agricultural sector rely on quantitative data such as farm income, land productivity, yield stability and farm efficiency. Within the extensive agricultural economics literature, two broad categories of economic indicators have been proposed. First, a group of simple economic indicators that includes profitability [3], farm revenues and household income [4] and crop yield [5] amongst others. Second, a group of more complex quantitative indicators which are calculated from several inputs and outputs such as farm productivity and technical efficiency. In this study, technical efficiency is used as a measure of farm economic sustainability. It is noteworthy to stress that technical efficiency is a necessary, but not sufficient condition for economic efficiency. The latter includes allocative efficiency as well [6]. Measurement of technical efficiency is relevant for at least two reasons. First, Improvement of technical efficiency is often the most cost-effective way of reducing inputs while keeping output levels constant. Second, policy-makers may find it easier to implement policies aimed at improving technical efficiency rather than introducing policies impacting the economic development.

While the literature discussed in-depth farm environmental indicators (including pesticides use, greenhouse gas emissions, biodiversity, water pollution, soil quality and land conservation), fewer studies exist on the social dimension of farm sustainability, in part due to the ambiguity surrounding the assessment of such issues [7–10]. While current definitions of sustainable agriculture focus mainly on environmental issues, a commonly accepted definition of the farm social sustainability dimension does not yet exist within the literature [11,12]. Some authors [13] suggest that social dimension concept integrates a set of indicators that can be classified into three on-farm objectives, namely education, working conditions and quality of life, and three off-farm objectives, namely agricultural practices that are acceptable from the society’s point of view and product quality. An example proposed for dairy farming [14] is a combination of farm working conditions and societal sustainability which includes food safety, animal welfare, and landscape quality. Another example is given in [15], with an assessment of the social sustainability of agricultural and regional communities which are affected by Coal Seam Gas in Australia. Using 5-point Likert type scales, the authors measure social dimension with the following items: access to healthy natural environment, access to infrastructure and economic opportunities, equity and governance, social cohesion, and community actualization. Despite the lack of consensus on the definition of farm social sustainability, as underlined in [16], the literature, however, agrees that social indicators can be classified in two categories: indicators related to the farm community (such as farmers’ working conditions, satisfaction and quality of life), and indicators associated to the broader society (such as vitality of rural areas and contribution to local employment). Measuring social dimension can be problematic as it is difficult to make these indicators objective and quantifiable. This is due to the dominant part of the subjective factors that affect social sustainability [17–19]. Self-reported qualitative measures, especially statements that are scored on a Likert scale, have been very practical for rating subjective factors within the sustainability literature but integrate the perception of the respondents [20–23].

Several papers have investigated the environmental and economic trade-offs of farms (e.g. [24–27]). But only a few papers address the trade-offs of farms between the three pillars of sustainability, economic, environmental and social. Among them, a sustainability investigation of coffee farms in Uganda [28] used the Sustainability Assessments of Food and Agriculture Systems (SAFA) framework elaborated by the Food and Agriculture Organization (FAO) to compute scores for various sub-themes within four main themes: economic resilience (e.g. investment, vulnerability), environmental integrity (e.g. water, biodiversity), social well-being (e.g. labour rights, human safety and health), and good governance (e.g. corporate ethics, holistic management). Trade-offs were assessed with correlations, showing synergies between social and governance sustainability, and between economic and environmental sustainability. Another example is [29], that considered farms in several countries of the European Union (EU) and clustered them on the basis of their economic performance, measured with one-dimension productivity and profitability indicators. Then, environmental and social performance of each cluster was assessed, using various computed environmental indicators (greenhouse gas emissions, nitrogen balance, water consumption, landscape elements, etc.) and indicators of farmers’ perceived quality of life, degree of stress and social engagement. The authors did not find a clear relationship between economic and environmental indicators, but showed that high social sustainability was associated with high economic performance. One can also mention [30] where the efficiency of Spanish farms is computed and decomposed it into classic technical efficiency, environmental efficiency and social efficiency using a non-parametric model with three sub-technologies. Environmental outputs were proxied by nitrogen and pesticide pollution, while social outputs were farmers’ measured injuries and farmers’ perceived satisfaction level. A positive association between environmental and social sustainability was shown, namely that environmentally sustainable farms tend to be operated by happier and healthier farmers. A recently study [31] used a production function based on a latent-class estimation procedure to account farm heterogeneity and shed light on the determinants of productivity and environmental sustainability performance. Weak trade-offs were found between productivity and environmental sustainability. Altough this paper investigated farm-level trade-offs, their study focuses on only mean-based environmental indicators and ignores social issues.

The objective of this article is to contribute to the literature on farms’ trade-offs between the three sustainability pillars. We aim to show that an approach that is currently used in the literature on economic and environmental trade-offs, can be applied to integrate the social dimension. More precisely, we use a stochastic frontier latent class model, which allows assessing technical efficiency of farms while accounting for differences in production technologies due to different environmental and social sustainability strategies. We apply this model to Spanish crop farms.

A growing literature is using the latent class stochastic frontier model to account for unobserved heterogeneity in agricultural production systems. This model allows the simultaneous estimation of farms’ efficiency and their statistical separation into different classes, using a number of separating variables. Most of the existing literature uses multi-dimensional indices as separating information to reflect the characteristics of the farm system, including feed per cow and stocking density [32], labor per cow and capital per cow [33], soil type [34] and share of grassland and agri-environmental subsidies [35]. The present work attempts to further extend this literature by addressing farms’ trade-offs between sustainability dimensions. In contrast to previous studies, we use self-reported information to capture differences across farms in terms of sustainability attitudes. Here we use information from multiple items to predict farmers’ commitment towards the environment and farmers’ contribution to on farm well-being and communities’ well-being. While the usefulness of qualitative data in measuring social issues has been previously established, several studies have argued that perception-based methods proved to be effective as well in generating environmental sustainability-related information [36,37].

The paper is organized as follows. The next section explains the methodology, and the third section describes the data and empirical specification. The fourth section presents the results and the last section concludes.

## 2. Technical efficiency and latent class analysis

Through the last decades, considerable research effort has been devoted to technical efficiency estimation, leading to significant contributions in both econometrics [38–41] and operational research literature on efficiency measurement [42–45]. One of the most commonly used parametric models is the stochastic frontier analysis (SFA) specified by Battese and Coelli [46] based on the original model of Aigner et al. [38]. The traditional stochastic frontier model assumes that all farms share the same technology. However, in practice, farms have heterogeneous production frontiers due to dissimilar technological levels [47], as well as differences in labour practices [48], environmental concerns [49], farm household’s structure [50], or management strategies [51]. Such discrepancies could lead to the use of different production technologies across farms. Since the standard SFA approach did not take into account such heterogeneity, erroneous estimations are likely to appear.

Different methods have been proposed to allow for heterogeneity in agricultural production literature. A commonly adopted technique is to split the sample (according to differences in e.g.: geographical distribution, main production, farmland ownership, conventional versus organic type) and estimate different frontiers [52–56]. The problem with this approach is that such a priori classification can be seen as an arbitrary decision. For example, farms that are in the same area or share the same production process, although belonging to the same group, may have different technologies simply because farmers may have different attitudes toward sustainability practices. Moreover, it is suggested that this procedure is not efficient because it does not use the information contained in one class to estimate the technology of other classes [57]. This interclass information can be very relevant because even though farms are classified into different groups, they may share some common underlying characteristics. Other researchers use cluster analysis to split the sample [58], while others used cluster analysis to identify heterogeneous farm groups according to their efficiency scores [59]. Further, other scholars [60–62] rely on different versions of random coefficient models in which farm heterogeneity is captured by continuous variation in technical parameters.

Another approach that has recently been recommended in the efficiency literature to deal with the unobserved heterogeneity of agricultural production in a robust way, is the stochastic frontier latent class model [57,60,63]. This approach separates the sample into several classes where each farm can be assigned to a specific class using class membership probabilities. The classification is done endogenously at the time of estimating the technical efficiency of farms.

For the *i*-th farm in class *j* (= 1,…,*J*), the finite mixture of several technologies writes as follows:

$$\ln\;Y_{i}=\beta_{j}\prime \;\ln\;X_{i}+v_{i|j}-u_{i|j}$$ (1)

where *Y*_*i*_ and *X*_*i*_ are respectively the output and input vector of the *i*-th farm; $v_{i|j}↝N(0,\sigma_{v|j}^{2})$ is the stochastic noise; *u*_*i*|*j*_ = |*U*_*i*|*j*_, $U_{i|j}↝N(0,\sigma_{u|j}^{2})$ is the technical inefficiency term which follows a half-normal distribution; and *β*_*j*_ is the class’ specific technological parameters.

The latent class stochastic frontier model is a single-stage approach which allows the probability for class membership and the mixture of technologies to be simultaneously estimated. The derived likelihood for each farm *i* is:

$${LF}_{i}(\theta ,\delta )=\sum_{j=1}^{J}{LF}_{j,i}{(\theta_{j})P}_{j,i}(\delta_{j})$$ (2)

with

$${0\leq P}_{j,i}(\delta_{j})=\frac{\exp\left(\delta_{j}^{\prime }q_{i}\right)}{\sum_{j=1}^{J}\exp\left(\delta_{j}^{\prime }q_{i}\right)}\leq 1$$ (3)

where the probability of belonging to a specific class is modelled as a multinomial logit function with *δ*_*J*_ = 0 which sets class *J* as the reference class, and ∑_*j*_*P*_*j*,*i*_(*δ*_*j*_) = 1; *q*_*i*_ is the vector of separating variables (to separate into classes) which are related to the latent problem.

The probability of farm *i* belonging to class *J* can be given by:

Pj,i(δj)=LFj,i(θj)=2σv|j2+σu|j2ϕ(ϵi|jσv|j2+σu|j2)Φ(−ϵi|j(σu|jσv|j)σv|j2+σu|j2)withϵi|j=vi|j−ui|j. (4)

Where *θ*_*j*_(.) represents the set of parameters of the likelihood function to be estimated for each class *J*.Using these estimated parameters, the posterior probabilities can be computed using the Bayes theorem to assign each farm to a particular class, namely the class with the highest posterior probability:

$$P(j|i)=\frac{{LF}_{j,i}({\hat{\theta }}_{j})P_{j,i}({\hat{\delta }}_{j})}{\sum_{j=1}^{J}{LF}_{j,i}({\hat{\theta }}_{j})P_{j,i}({\hat{\delta }}_{j})}$$ (5)

Technical efficiency is assumed to have values between 0 and 1, and is defined as the farm’s ability to produce the highest level of output from a given level of inputs. In our study, we consider it as the proxy for economic sustainability. In the classical stochastic frontier model where all farms share the same technology, predictions of conditional technical efficiency (TE) are calculated according to the following expression [64]:

$$E_{i}=exp(-u_{i}|\epsilon_{i})$$ (6)

The latent class stochastic production frontier model takes into account the underlying heterogeneity among farms by endogenously separating them into a number of classes. For each class *j*, a different production frontier is estimated, and the probability of belonging to each class is estimated for each farm, therefore, technical efficiency is estimated for each class. Following [57], it is measured using the weighted average of the technical efficiencies for all the frontiers considered with the corresponding (posterior) class probabilities as weights:

$$E_{i}=\sum_{j=1}^{J}P(j/i)∙E_{i|j}$$ (7)

While some literature applying the latent stochastic frontier model to farms aimed at identifying technologies based on labour or capital intensity [33,34], it has been also used to consider economic and environmental sustainability [32,35]. In these studies, the economic sustainability is proxied by the dependent variable technical efficiency, while environmental aspects are captured through separating variables representing farms’ environmental commitments such as environmentally-friendly practices applied on the farm. In our study, we integrate an additional aspect, namely social sustainability captured through separating variables representing farms’ social commitments.

The empirical model of the production frontier with a single-output Cobb-Douglas specification is expressed as follows:

$$lnY_{i}=\beta_{0|j}+\beta_{c|j}\;ln\;c_{i}+\beta_{r|j}\;ln\;r_{i}+\beta_{1|j}\;ln\;x_{1i}+\beta_{2|j}\;ln\;x_{2i}+\beta_{3|j}\;ln\;x_{3i}+\beta_{4|j}\;ln\;x_{4i}+v_{i|j}-u_{i|j}$$ (8)

It is important to note that the parameters of the latent classes and their efficiency scores are simultaneously estimated with model (8) using a single-stage approach, where *β*_0_ represents the intercept term; *β*_*k*|*j*_ (*k* = *c*, *r*, 1, 2, 3, 4) is a vector of parameters to be estimated for each class *j*. The two components *v*_*i*_ and *u*_*i*_ are assumed to be mutually independent.

The estimation of Eq (7) was carried out using the ‘ucminf” R-package [65]. Multiple indicators can be used to identify the appropriate number of classes. In addition, the presence of potential over-specification, such as imprecise parameters and groups with a reduced number of individuals, should also be taken into consideration when selecting the appropriate number of classes [66]. Here, the information criteria such as the Akaike information criterion (AIC) and the Bayesian information criterion (BIC) [67] were used as selection process by choosing the model with the smallest information criteria. Both statistics favour the model with two classes against three and four latent classes models, which is consistent with the distribution of farms across the two classes (71 and 109 farms). Results are presented and discussed in the next section.

## 3. Data and empirical specification

We apply the latent stochastic frontier model on farm-level data collected through a specific survey in 2015 from a sample of 180 Spanish farms specialized in the production of cereal, oilseed and protein (COP) crops and located in the region of Catalonia in Spain. The data were collected through face-to-face interviews by specialist technicians from Unió de Pagesos (UDP), the largest farmer association in Catalonia. All farmers agreeing to take part in the survey were asked to sign a consent form, in which they agreed to participate in the research project. A farm is considered as specialized in COP crop production, when revenue from COP crops represents at least 80% of the farm total income. With the condition of at least 80% of overall farm revenues are generated from COP, around 250 agricultural holdings have been identified as meeting the specialization criteria from a large list of 7000 farms. From there, 180 farms have been surveyed.

Table 1 provides summary statistics for the output (*Y*), inputs (*X*) and separating variables (*q*) considered in this study.

**Table 1:** Descriptive statistics for the variables used in the latent class analysis.

	Variables	Unit	Mean	St. Dev.
Output	*Y*			
Crop production	*y*	Kilograms	373,148	359,489
Inputs	*X*			
Pesticides	*c*	Litres	103.4	149.7
Nitrogen	*r*	Kilograms	10,055	11,227
Crop land area	*x*_1_	Hectares	80.7	73.6
Capital	*x*_2_	Euros	157,837	169,036
Total labour	*x*_3_	Hours	827.1	841.9
Energy	*x*_4_	Euros	4,935	5,199
Separating variables	*q*			
Environmental commitment	*q*_1_	Categorical	505.0	74.2
Farmers’ well-being	*q*_2_	Categorical	493.8	67.8
Local communities’ well-being	*q*_3_	Categorical	483.8	62.3

In the production function specification (see model (8)), the output is represented by the farm crop production measured in kilograms, which is about 373 thousand kilogrammes on average. Due to missing price information, we are only able to show the aggregate output quantities. However, this aggregation does not necessarily represent a methodological challenge, since our crops are positively and significantly correlated. The inputs used are: two crop-specific inputs, pesticides use and nitrogen use. As regard pesticides use (*c* in litres), data collected through the survey included detailed information on herbicides, fungicides and insecticides application, including the chemical formula. In addition to this collected information, densities were used to express the total amount of active ingredients in liters. As regard nitrogen use (*r* in kilograms), the data also included details on chemical and organic fertilizers applied by farmers. The amount of nitrogen contained in chemical fertilizers can be computed from this information. The estimation of the nitrogen quantity contained in organic fertilizers and seeds was less straightforward, hence we relied on external information provided in [68] to estimate the amount of nitrogen present in organic fertilisers and coefficients provided by Spanish Ministry of Agriculture [69] to approximate the quantities of nitrogen content in seeds.

The other inputs are: crop land area (*x*_1_ in hectares); capital replacement value (*x*_2_ in Euros); total labour, both paid and unpaid (*x*_3_ in hours); and energy (*x*_4_ in Euros). On average, the sample farms annually apply 103.4 litres of pesticides, while nitrogen application slightly exceeds 10 thousand kilograms per year. On average they cultivate 80.7 hectares of crops, have a capital value of around 157 thousand Euros, use 827.1 labour hours per year on the farm and spend 4,935 Euros on energy.

The separating variables proxy the degree of environmental sustainability on the one hand, and of social sustainability, on the other hand. They rely on farmers’ attitudes and commitment. The information was collected using a 4-point Likert scale, and farmers were asked to rate 12 statements that are related to environmental and social responsibility (see Table 2). This data collection is based on farmers’ subjective perceptions, which might lead to identification biases. Some procedures have been proposed by the literature to deal with this issue, such as the use of “cheap-talk” [70]. However, other studies, such as [71] have shown that these techniques may be ineffective with experienced participants. Furthermore, the fact that interviewers were technicians from Unió de Pagesos who know the farm well, is expected to have reduced farmers’ incentives to provide biased responses.

**Table 2:** Sustainability statements used to define the three separating variables.

Separating variable		Statement
Environmental commitment (*q*_1_)	S1	Agricultural activities of my farm contribute positively to the landscape quality.
	S2	Our agricultural activities contribute to the diversification and/or preservation of fauna and flora.
Farmers’ well-being (*q*_2_)	S3	In the farm work, schedules are flexible.
	S4	The number of holidays that I have is enough.
	S5	I find my job to be motivating.
	S6	I am satisfied with work and working conditions in the farm.
Local communities’ well-being (*q*_3_)	S7	Our farm products are safe for consumers’ health.
	S8	Products from the farm contribute to food security in the region.
	S9	Our farm contributes positively to the local economy.
	S10	Our farm contributes to the social fabric of rural communities.
	S11	Our farm contributes to maintain basic services (schools. health facilities, etc.…) in rural areas.
	S12	Our farm helps reducing local unemployment.

Note: farmers had to rate each statement along a 4-point Likert scale: ‘Strongly disagree’, ‘Disagree’, ‘Agree’, ‘Strongly agree’.

More specifically, the 12 statements are grouped into three categories: environmental commitment (*q*_1_), which consists of two statements; farmers’ well-being (*q*_2_), which contains 6 statements; local communities’ well-being (*q*_3_), which is reflected by a set of 4 statements. Environmental sustainability is proxied by farmers’ attitudes and commitment towards landscape (S1 in Table 2) and biodiversity (S2). Social sustainability comprises two themes, namely private social sustainability and public social sustainability. The former consists of farmers’ appreciation of their well-being, in terms of workload (S3 and S4) and work satisfaction (S5 and S6). The latter is related to the farm’s involvement in the local community, in terms of food safety and security (S7 and S8), and rural areas’ vitality (S9, S10, S11 and S12).

Following standard practice (for examples, see [72], each of the three separating variables consists of the 4-point Likert scale statements whose scores range on a scale of 25–100: strongly disagree (25), disagree (50), agree (75), strongly agree (100). For each separating variable, the scores of all statements were added, and the resulting variables were standardized to have similar magnitudes since they do not include the same number of statements: the separating variable ‘Environmental commitment’ was multiplied by 3 and the variable ‘Farmers’ well-being’ was multiplied by 1.5. Following this, all three separating variables have a maximal score of 600, with increasing score values denoting higher levels of sustainability. As suggested by one anonymous reviewer, we have constructed the separating variables (*q*_1_, *q*_2_, *q*_3_) using an alternative method, namely Principal Component Analysis (PCA). After performing PCA on the groups of items used to construct our separating variables, we find that the first Principal Component of each group of items is positively and highly correlated with the corresponding separating variable (as we originally constructed and used them in the paper). We do not perform the empirical application with this alternative measure because we assess that it fails to capture relevant aspects of the data.

## 4. Results

The estimated results of the stochastic frontier latent class model are shown in Table 3. The second part of the table displays the effect of the separating variables on the probability to belong to the first class, shown in the first columns of Table 3. The coefficients of the three separating variables are significant. The negative sign of the coefficients for ‘Farmers’ well-being’ (*q*_2_) and ‘Local communities’ well-being’ (*q*_3_) indicates that a higher value of these variables increases the probability of a farm not ending up in the first class, while the coefficient ‘Environmental commitment’ (*q*_1_) has a positive sign, implying that a higher value of this variable increases the probability that a farm belongs to the second class. Hence, farms of this class have a higher perception of environmental sustainability, while farms in the other class have a higher perception of social sustainability. We will denote both classes ‘Environmentally sustainable farms’ and ‘Socially sustainable farms’, respectively. Recall that both classes reflect farmers’ perception towards environmental and social sustainability. This separation based on the coefficients of the separating variables clearly shows the existence of trade-offs between environmental sustainability and social sustainability.

**Table 3:** Latent class model parameter estimates.

	Class 1: Environmentally sustainable farms (71 farms)			Class 2: Socially sustainble class (109 farms)		
	Estimate	Standard Error	P-value	Estimate	Standard Error	P-value
**Production function**						
Intercept	8.20482649	2.22E-04	0.000	8.70753633	3.16E-01	0.000
Pesticides	0.05370777	2.17E-05	0.000	0.02539479	1.37E-02	0.063
Nitrogen	0.18819716	2.28E-05	0.000	-0.00519721	4.44E-02	0.907
Crop land area	0.74435555	6.44E-05	0.000	1.10314188	6.53E-02	0.000
Capital	0.00259175	3.18E-05	0.000	0.00170819	7.81E-03	0.827
Total labour	0.07778004	4.32E-05	0.000	-0.08695737	3.53E-02	0.014
Energy	-0.09743686	4.97E-05	0.000	0.00266691	4.29E-02	0.950
Sigma_u	0.2700418	1.52E-01	0.075	0.09422609	3.73E-01	0.800
Sigma_v	9.4816E-15	2.75E+02	1.000	0.01312151	6.83E-01	0.985
**Probabilities to belong to class 1**						
Intercept	4.355987	6.09E+00	0.475			
Environmental commitment	0.04958766	1.58E-02	0.002			
Farmers’ well-being	-0.02262501	1.11E-02	0.042			
Local communities’ well-being	-0.03936784	1.52E-02	0.009			

Based on the estimated posterior probabilities of class membership, 71 farms were classified as environmentally sustainable farms, 109 farms were classified as socially sustainable farms. It is worth mentioning that the heterogeneity of the sample farms which is associated with environmental and social sustainability is quite obvious, as the average posterior probability of being environmentally sustainable (respectively, socially sustainable) of those farms classified in the environmentally sustainable (respectively, socially sustainable) class, was very large, namely 85.8% (respectively 87.7%). Further, having a different number of farms across the two classes would indicate that there is indeed a trade-off between farmers who are committed to the environment and farmers who are commited to improve their well-being.

**Fig 1:**
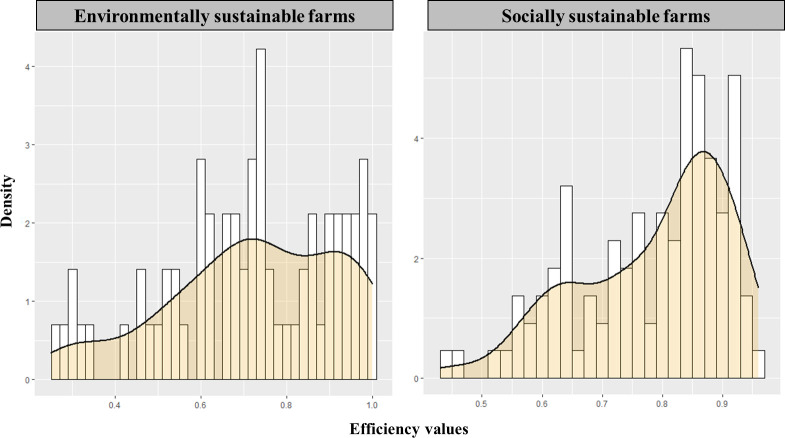
Distribution of technical efficiency scores with an overlaid kernel density estimate for each class.

Fig 1 presents histograms of the technical efficiency distributions and nonparametric kernel density functions for each class. The left-skewed distribution for the socially sustainable farms’ class indicates that many farms in this class are located close to the efficient frontier. More precisely, three quarters of the farms have a score greater than 0.7. The environmentally sustainable farms’ class shows a flatter distribution with a much less pronounced peak than the other class, with more than 40% of the sample having a technical efficiency score below 0.7. Fig 1 clearly illustrates differences in terms of technical efficiency between farms that strongly consider social issues and farms that are more concerned by environmental issues. This is confirmed by the difference in average technical efficiency between both classes: the environmentally sustainable farms have an average technical efficiency of 0.72 while the figure is 0.78 for socially sustainable farms, a difference which is significant (see Table 4). This suggests that farms face trade-offs, not only between environmental and social sustainability, but also with economic sustainability (technical efficiency). Achieving high technical efficiency is more difficult when contributing to environmental sustainability than when contributing to social sustainability. Putting more effort into reducing resource use and protecting the environment is likely to lead to a decline in technical efficiency, while technical efficiency improves when the interests of the various stakeholders (including farmers, their families, on farm workers, and the local community) are better balanced. This finding is consistent with the notion of rational efficiency [73]. Following this notion, one might interpret the relativly low technical efficiency levels of both classes as a result of farmers rational decisions, which most likely place a greater weights on environmental and social issues than on higher economic performance. This is compatible with rational inefficiency explanation suggested by [74] in which Swedish farmers apply relatively high levels of animal welfare measures to the extent that it reduces their technical efficiency.

**Table 4:** Descriptive statistics for environmental sustainable farms and socially sustainable farms.

	Units	Environmentally sustainable class (71 farms)		Socially sustainable class (109 farms)		T-test	
		Mean	St. Dev.	Mean	St. Dev.	t-value	p-value
Technical efficiency		0.72	0.20	0.78	0.12	**-2.34**	**0.02**
Environmental commitment	Categorical	545.1	53.7	478.9	74.2	**6.93**	**0.00**
Farmers’ well-being	Categorical	468.0	69.1	510.6	61.6	**- 4.21**	**0.00**
Local communities’ well-being	Categorical	470.4	61.3	492.4	61.7	**- 2.35**	**0.02**
Crop production	Kilograms	334,156	268,844	398,547	407,055	- 1.28	0.20
Pesticides	Litres	94.7	112.9	109.0	169.6	- 0.68	0.50
Nitrogen	Kilograms	8,901	9,222	10,806	12,343	-1.18	0.24
Crop land area	Hectares	79.6	63.2	81.3	79.9	- 0.16	0.88
Capital	Euros	158,372	193,171	157,488	152,222	0.03	0.97
Total labour	Hours	840.5	785.3	818.4	880.2	0.18	0.86
Energy	Euros	5,549	5,453	4,536	5,012	1.26	0.21
Age	Years	53.6	10.3	52.5	13.1	0.65	0.52
Education level	Categorical	2.72	0.61	2.67	0.59	0.53	0.60
Farmer’s experience in agriculture	Years	34.9	13.7	32.6	15.2	1.02	0.31
CAP subsidies per hectare	Euros	180.09	69.44	190.19	153.76	-0,6	0,55
CAP subsidies per kg crop	Euros	0.052	0.05	0.04	0.05	1,10	0,26
CAP subsidies per hour worked	Euros	24.53	19.54	25.12	23.48	-0,18	0,85

Note: Education level is measured as a five categories variable, corresponding to ‘Not received any education’ (1), ‘Primary education only’ (2), ‘Secondary education only’ (3), ‘University education lower than PhD’ (4), and ‘PhD level’ (5).

Going back to Table 3, the first part of the table provides the estimated stochastic production frontier parameters for farms in both classes. The coefficients of all input variables are significant and have expected positive effects on agricultural output in the case of the environmentally sustainable farms, except for energy whose coefficient has a negative significant sign. Regarding the socially sustainable farms, expected significant positive effects are found for land and pesticides, while nitrogen, capital and energy have no significant impact and total labour has a negative significant impact. The negative effect of energy on the output for environmental sustainable farms may come from the fact that these farms over-use energy when replacing chemical treatments by mechanical treatment. As for the negative coefficient of labour for the socially sustainable farms, several authors [75–77] also found a negative sign for family farms’ labour. This might be because family farms have been typically characterized by very little hired labour, in turn implying excess labour hours by family members.

To understand further the discrepancy in the production function between both classes, an analysis of mean differences in terms of technical efficiency scores, output, inputs, separating variables and additional variables was conducted between the obtained two classes using t-tests. The additional variables considered relate to some farms’ and farmers’ features: farmers’ age, farmer’s education level, farmer’s number of years in experience in agriculture, and amount of subsidies received by the farms in the frame of the EU CAP. The results are presented in Table 4.

We find significant mean differences between the two classes in terms of technical efficiency score, as mentioned above, and the three separating variables (first four rows in Table 4). Intuitively, environmentally sustainable farms show a significantly higher average score of 545.1 (vs. 478.9) in environmental commitment, while the class of socially sustainable farms shows significantly better performance in terms of accounting for farmers’ well-being (510.6 vs. 468.0) and, to a lesser extent, for communities’ well-being (492.4 vs. 470.4).

This is further confirmed when analysing mean differences between both classes in terms of inputs use and output production (Table 4). Although the differences are not significant, the patterns are consistent, since the socially sustainable farms produce on average 64 thousands kilograms of crop output more than environmentally sustainable farms, while the latter show a lower use of polluting inputs, with almost 15 litres of pesticides and 1.9 thousand kilograms of nitrogen lower than socially sustainable farms. Environmentally sustainable farms are expected to limit the use of pesticides and fertilizers since such inputs could lead to ecological hazards [78], while socially sustainable farms are expected to reach higher level of output since larger revenue allow farmers to pay themselves a fair market wage, which undoubtedly supports the local economy and farming communities. In addition, environmentally sustainable farms use higher labour and energy to carry out mechanical operations instead of chemical operations, which may be responsible for technical efficiency degradation.

The last six rows in Table 4 show the mean comparison between both classes in terms of additional farms’ and farmers’ characteristics. Both classes do not differ in farmers’ socio-demographic characteristics (age, education, experience), suggesting that none of these factors are behind farmers’ heterogeneity in term of their attitude towards environmental and social sustainability. This is in line with studies that have tried to shed light on the relationship between farmer demographic characteristics and sustainable practices, whose results have been inconsistent or contradictory [79]. As for the level of CAP subsidies received by farms, it does not differ significantly across classes, in line with the results of [29] that subsidies were not consistently related to economic nor environmental performance.

Based on the results of the latent class model, the MEO (maximal expected output) can be calculated through the equation $MEO=f(x_{n|j}^{o},\beta_{n|j})$, where $x_{n|j}^{o}$ is the observed input and *β*_*n*|*j*_ is the corresponding estimated parameter. For comparison, the MEO based on the pooled model is also computed. The results reveal that socially sustainable farms have a higher potential output (497,848 kg) in comparison with environmentally sustainable farms (481,306 kg). The results also suggest that the MEO based on the pooled model where all farms share the same technology would overestimate farms’ potential output production (506,600 kg), confirming that heterogeneous production frontiers related to environmental and social sustainability exist within our sample of farms.

## 5. Conclusion

In this article, we present an innovative approach to study trade-offs of farms in terms of economic sustainability (proxied here by technical efficiency), environmental sustainability (proxied here by farmers’ pro-environmental attitudes and commitments) and social sustainability (proxied here by farmers’ contribution to on farm well-being and communities well-being). We used the latent class stochastic frontier model which enables accounting for unobserved heterogeneity, estimating simultaneously specific production functions and farms’ technical efficiency for different classes. Here, classes were obtained based on three separating variables, representing farms’ environmental sustainability and social sustainability, derived from statements scored by farmers. The application was to a sample of Spanish crop farms in 2015.

Our findings show that the more environmentally sustainable farms are, the less likely they are to operate at a high level of technical efficiency, which is in line with previous findings that environmentally friendly practices can affect farm productivity and efficiency [80–82]. Our results suggest, however, that improvements in social concerns, both towards own farm and the larger community, may lead to improved technical efficiency level. This is compatible with the argument that economic benefits are linked to social issues since income and financial wealth are prerequisites for accessing better social opportunities [83].

In general, our study provides evidence of existing trade-offs for farms between economic sustainability and environmental sustainability, but also between environmental sustainability and social sustainability. The latter finding suggests that policies should be designed to help environmentally sustainable farms achieve decent levels of private social sustainability. Switching from chemical protection of crops to mechanical practices for example may indeed be unfavourable to farmers’ working conditions.

A methodological limit is that here social sustainability and environmental sustainability were assessed subjectively, with farmers’ perceived contribution of their farm to these dimensions. In terms of environmental aspects, such approach may avoid errors or approximation in the measurement of specific environmental outputs such as greenhouse gases, landscape elements etc. However, self-reporting of contribution may differ from actual contribution. Although descriptive statistics of our environmentally sustainable farms show that these farms use fewer chemical inputs, this does not fully support farmers’ scoring of statements about environmental commitment. As in many other studies, social sustainability was assessed here with farmers’ perception. This is less problematic as for environmental sustainability, since for private (own) social sustainability such as working conditions or satisfaction, farmers are the ones who know better and thus their statements scoring can be trusted. As regards social sustainability related to the wider community, statements may not totally reflect the reality, but what is interesting from our study is that, when farmers think that they contribute to the community’s sustainability, then it increases their economic sustainability. Furthermore, it should be noted that we are using a binary classification to distinguish between environmental sensitivity and social responsibility, while both concepts tend to overlap [84]. Our study is therefore a first application of the latent class modelling framework to the integration of social aspects, and further empirical applications are needed in particular to suggest improvements in the way sustainability indicators are measured.

Our analysis focused on a specific year and should be replicated over a longer period. Results may differ when several years are considered, with increased trade-offs in sustainability dimensions in case of e.g. economic, climate, pest or farmer’s health shocks. By contrast, trade-offs may be reduced over time. As underlined by [26], in the longer term, environmentally friendly practices may result in more stable yields and reduced risk. In methodological terms, this could be done by assessing the persistence degree in each class or the switch between classes.

## References

[1] Piñeiro V., Arias J., Dürr J., Elverdin P., Ibáñez A.M., Kinengyere A., et al. A scoping review on incentives for adoption of sustainable agricultural practices and their outcomes. Nat. Sustain. 3, 809–820. 10.1038/s41893-020-00617-y.

[2] NRC, 2010. Toward Sustainable Agricultural Systems in the 21st Century. Washington, D.C. National Academies Press. 10.17226/12832.

[3] Testa R., Foderà M., Di Trapani A.M., Tudisca S., Sgroi F., 2015. Choice between alternative investments in agriculture: The role of organic farming to avoid the abandonment of rural areas. Ecol. Eng. 83, 227–232. 10.1016/j.ecoleng.2015.06.021.

[4] Chopin P., Doré T., Guindé L., Blazy J.M., 2015. MOSAICA: A multi-scale bioeconomic model for the design and ex ante assessment of cropping system mosaics. Agric. Syst. 140, 26–39. 10.1016/j.agsy.2015.08.006.

[5] Sydorovych O., Raczkowski C.W., Wossink A., Mueller J.P., Creamer N.G., Hu S., et al. A technique for assessing environmental impact risks of agricultural systems. Renew. Agric. Food Syst. 24, 234–243. 10.1017/S174217050999010X.

[6] Kalirajan K.P., 1990. On measuring economic efficiency. J. Appl. Econom. 5, 75–85. 10.1002/jae.3950050106.

[7] Dempsey N., Bramley G., Power S., Brown C., 2011. The social dimension of sustainable development: Defining urban social sustainability. Sustain. Dev. 19, 289–300.

[8] Murphy K., 2012. The social pillar of sustainable development: a literature review and framework for policy analysis. Sustain. Sci. Pract. Policy 8.

[9] Seager T.P., 2008. The sustainability spectrum and the sciences of sustainability. Bus. Strateg. Environ. 17, 444–453. 10.1002/bse.632.

[10] Zahm F., Viaux P., Vilain L., Girardin P., Mouchet C., 2008. Assessing farm sustainability with the IDEA method—from the concept of agriculture sustainability to case studies on farms. Sustain. Dev. 16, 271–281. 10.1002/sd.380.

[11] Chambers R.G., Serra T., 2018. The social dimension of firm performance: a data envelopment approach. Empir. Econ. 54, 189–206. 10.1007/s00181-016-1135-z.

[12] Allen P., Van Dusen D., Lundy J., Gliessman S., 1991. Integrating social, environmental, and economic issues in sustainable agriculture. Am. J. Altern. Agric. 6, 34. 10.1017/S0889189300003787.

[13] Lebacq T., Baret P. V, Stilmant D., 2013. Sustainability indicators for livestock farming. A review. Agron. Sustain. Dev. 33, 311–327.

[14] Van Calker K.J., Berentsen P.B.M., De Boer I.J.M., Giesen G.W.J., Huirne R.B.M., 2007. Modelling worker physical health and societal sustainability at farm level: an application to conventional and organic dairy farming. Agric. Syst. 94, 205–219.

[15] Phelan A. (Anya), Dawes L., Costanza R., Kubiszewski I., 2017. Evaluation of social externalities in regional communities affected by coal seam gas projects: A case study from Southeast Queensland. Ecol. Econ. 131, 300–311. 10.1016/J.ECOLECON.2016.09.010.

[16] Diazabakana A., Latruffe L., Bockstaller C., Desjeux Y., Finn J., Kelly E., et al. A Review of Farm Level Indicators of Sustainability with a Focus on CAP and FADN. Available online: https://www.flint-fp7.eu/downloads/reports/FLINT%20WP1%20_D1_2.pdf 20.

[17] Edum-Fotwe F.T., Price A.D.F., 2009. A social ontology for appraising sustainability of construction projects and developments. Int. J. Proj. Manag. 27, 313–322. 10.1016/J.IJPROMAN.2008.04.003.

[18] Boström M., 2012. The problematic social dimension of sustainable development: the case of the Forest Stewardship Council. Int. J. Sustain. Dev. World Ecol. 19, 3–15. 10.1080/13504509.2011.582891.

[19] Dillard J., Dujon V., King M.C., 2008. Understanding the social dimension of sustainability In: Dillard J, Dujon V, King MC, editors. Understanding the social dimension of sustainability. Routledge, London (UK).

[20] Duke J.M., Borchers A.M., Johnston R.J., Absetz S., 2012. Sustainable agricultural management contracts: Using choice experiments to estimate the benefits of land preservation and conservation practices. Ecol. Econ. 74, 95–103. 10.1016/j.ecolecon.2011.12.002.

[21] Garmendia E., Stagl S., 2010. Public participation for sustainability and social learning: Concepts and lessons from three case studies in Europe. Ecol. Econ. 69, 1712–1722. 10.1016/j.ecolecon.2010.03.027.

[22] Vermeir I., Verbeke W., 2008. Sustainable food consumption among young adults in Belgium: Theory of planned behaviour and the role of confidence and values. Ecol. Econ. 64, 542–553. 10.1016/j.ecolecon.2007.03.007.

[23] Yigitcanlar T., Dur F., 2010. Developing a sustainability assessment model: The sustainable infrastructure, Land-use, environment and transport model. Sustainability 2, 321–340. 10.3390/su2010321.

[24] Berre D., Blancard S., Boussemart J.P., Leleu H., Tillard E., 2014. Finding the right compromise between productivity and environmental efficiency on high input tropical dairy farms: A case study. J. Environ. Manage. 146, 235–244. doi: 10.1016/j.jenvman.2014.07.008 25178529

[25] Haggar J., Soto G., Casanoves F., Virginio E. de M., 2017. Environmental-economic benefits and trade-offs on sustainably certified coffee farms. Ecol. Indic. 79, 330–337. 10.1016/j.ecolind.2017.04.023.

[26] Rosa-Schleich J., Loos J., Mußhoff O., Tscharntke T., 2019. Ecological-economic trade-offs of Diversified Farming Systems–A review. Ecol. Econ. 10.1016/j.ecolecon.2019.03.002.

[27] Villalba D., Díez-Unquera B., Carrascal A., Bernués A., Ruiz R., 2019. Multi-objective simulation and optimisation of dairy sheep farms: Exploring trade-offs between economic and environmental outcomes. Agric. Syst. 173, 107–118. 10.1016/j.agsy.2019.01.011.

[28] Ssebunya B.R., Schader C., Baumgart L., Landert J., Altenbuchner C., Schmid E., Stolze M., 2019. Sustainability Performance of Certified and Non-certified Smallholder Coffee Farms in Uganda. Ecol. Econ. 156, 35–47. 10.1016/j.ecolecon.2018.09.004.

[29] Latruffe L., Desjeux Y., Hanitravelo G.L.J., Hennessy T., Bockstaller C., Dupraz P., Finn J., 2016. Tradeoffs between economic, environmental and social sustainability: The case of a selection of European farms. EU FP7 FLINT (Farm-Level Indicators for New Topics in policy evaluation), Deliverable 5.2L. 29 December. 1–46.

[30] Ait Sidhoum A., Serra T., Latruffe L., 2020. Measuring sustainability efficiency at farm level: A data envelopment analysis approach. Eur. Rev. Agric. Econ. 47, 200–225. 10.1093/erae/jbz015.

[31] Sauer J., Moreddu C., 2020. Drivers of farm performance:: Empirical country case studies, OECD Food, Agriculture and Fisheries Papers. Paris.

[32] Alvarez A., Del Corral J., 2010. Identifying different technologies using a latent class model: Extensive versus intensive dairy farms. Eur. Rev. Agric. Econ. 37, 231–250. 10.1093/erae/jbq015.

[33] Sauer J., Morrison Paul C.J., 2013. The empirical identification of heterogeneous technologies and technical change. Appl. Econ. 45, 1461–1479. 10.1080/00036846.2011.617704.

[34] Martinez Cillero M., Thorne F., Wallace M., Breen J., 2019. Technology heterogeneity and policy change in farm-level efficiency analysis: An application to the Irish beef sector. Eur. Rev. Agric. Econ. 46, 193–214. 10.1093/erae/jby028.

[35] Dakpo K. H., Latruffe L., Desjeux Y., Jeanneaux P., 2021. Latent Class Modelling for a Robust Assessment of Productivity: Application to French Grazing Livestock Farms. J. Agric. Econ. 72, 760–781. 10.1111/1477-9552.12422.

[36] Rahman S., 2003. Environmental impacts of modern agricultural technology diffusion in Bangladesh: An analysis of farmers’ perceptions and their determinants. J. Environ. Manage. 68, 183–191. doi: 10.1016/s0301-4797(03)00066-5 12781758

[37] Sabiha N.E., Salim R., Rahman S., Rola-Rubzen M.F., 2016. Measuring environmental sustainability in agriculture: A composite environmental impact index approach. J. Environ. Manage. 166, 84–93. doi: 10.1016/j.jenvman.2015.10.003 26492465

[38] Aigner D., Lovell C.A.K., Schmidt P., 1977. Formulation and estimation of stochastic frontier production function models. J. Econom. 6, 21–37. 10.1016/0304-4076(77)90052-5.

[39] Battese G.E., Corra G.S., 1977. Estimation of a production frontier model: with application to the pastoral zone of eastern australia. Aust. J. Agric. Econ. 21, 169–179. 10.1111/j.1467-8489.1977.tb00204.x.

[40] Jondrow J., Knox Lovell C.A., Materov I.S., Schmidt P., 1982. On the estimation of technical inefficiency in the stochastic frontier production function model. J. Econom. 19, 233–238. 10.1016/0304-4076(82)90004-5.

[41] Meeusen W., van Den Broeck J., 1977. Efficiency Estimation from Cobb-Douglas Production Functions with Composed Error. Int. Econ. Rev. (Philadelphia). 18, 435. 10.2307/2525757.

[42] Banker R.D., Charnes A., Cooper W.W., 1984. Some Models for Estimating Technical and Scale Inefficiencies in Data Envelopment Analysis. Manage. Sci. 30, 1078–1092. 10.1287/mnsc.30.9.1078.

[43] Charnes A., Cooper W.W., Rhodes E., 1978. Measuring the efficiency of decision making units. Eur. J. Oper. Res. 2, 429–444.

[44] Färe R., Grosskopf S., Lovell C.A.K., 1985. The Measurement of Efficiency of Production. Springer Netherlands, Dordrecht, pp. 1–199. 10.1007/978-94-015-7721-2_1.

[45] Farrell M.J., 1957. The measurement of productive efficiency. J. R. Stat. Soc. Ser. A 120, 253–290.

[46] Battese G.E., Coelli T.J., 1995. A model for technical inefficiency effects in a stochastic frontier production function for panel data. Empir. Econ. 20, 325–332. 10.1007/BF01205442.

[47] Zhengfei G., Oude Lansink A., 2006. The Source of Productivity Growth in Dutch Agriculture: A Perspective from Finance. Am. J. Agric. Econ. 88, 644–656. 10.1111/j.1467-8276.2006.00885.x.

[48] Ahituv A., Kimhi A., 2005. Simultaneous estimation of work choices and the level of farm activity using panel data. Eur. Rev. Agric. Econ. 33, 49–71. 10.1093/erae/jbi035.

[49] Upton M., Haworth S., 1987. The growth of farms. Eur. Rev. Agric. Econ. 14, 351–366. 10.1093/erae/14.4.351.

[50] Chang H.-H., Yen S.T., 2010. Off-farm employment and food expenditures at home and away from home. Eur. Rev. Agric. Econ. 37, 523–551. 10.1093/erae/jbq032.

[51] Huijps K., Hogeveen H., Antonides G., Valeeva N.I., Lam T.J.G.M., Oude Lansink A.G.J.M., 2010. Sub-optimal economic behaviour with respect to mastitis management. Eur. Rev. Agric. Econ. 37, 553–568. 10.1093/erae/jbq036.

[52] Bravo-Ureta B.E., Evenson R.E., 1994. Efficiency in agricultural production: The case of peasant farmers in eastern Paraguay. Agric. Econ. 10, 27–37. 10.1016/0169-5150(94)90037-X.

[53] Chen Z., Huffman W.E., 2009. Farm technology and technical efficiency: Evidence from four regions in China. China Econ. Rev. 20, 153–161. 10.1016/J.CHIECO.2009.03.002.

[54] Guesmi B., Serra T., Kallas Z., Gil Roig J.M., 2012. The productive efficiency of organic farming: the case of grape sector in Catalonia. Spanish J. Agric. Res. 10, 552. 10.5424/sjar/2012103-462-11.

[55] Latruffe L., Balcombe K., Davidova S., Zawalinska K., 2004. Determinants of technical efficiency of crop and livestock farms in Poland. Appl. Econ. 36, 1255–1263. 10.1080/0003684042000176793.

[56] Manjunatha A.V., Anik A.R., Speelman S., Nuppenau E.A., 2013. Impact of land fragmentation, farm size, land ownership and crop diversity on profit and efficiency of irrigated farms in India. Land use policy 31, 397–405. 10.1016/J.LANDUSEPOL.2012.08.005.

[57] Orea L., Kumbhakar S.C., 2004. Efficiency measurement using a latent class stochastic frontier model. Empir. Econ. 29, 169–183. 10.1007/s00181-003-0184-2.

[58] Alvarez A., del Corral J., Solís D., Pérez J.A., 2008. Does intensification improve the economic efficiency of dairy farms? J. Dairy Sci. 91, 3693–8. doi: 10.3168/jds.2008-1123 18765628

[59] Bojnec Š., Latruffe L., 2008. Measures of farm business efficiency. Ind. Manag. Data Syst. 108, 258–270. 10.1108/02635570810847617.

[60] Greene W., 2005. Reconsidering heterogeneity in panel data estimators of the stochastic frontier model. J. Econom. 126, 269–303. 10.1016/J.JECONOM.2004.05.003.

[61] Huang H. (river), 2004. Estimation of Technical Inefficiencies with Heterogeneous Technologies. J. Product. Anal. 21, 277–296. 10.1023/B:PROD.0000022094.39915.cf.

[62] Kalirajan K.P., Obwona M.B., 1994. Frontier Production Function: The Stochastic Coefficients Approach. Oxf. Bull. Econ. Stat. 56, 87–96.

[63] Greene W., 2002. Alternative panel data estimators for stochastic frontier models. Unpubl. Manuscr. (Septemebr 1, 2002), Dep. Econ. New York Univ.

[64] Battese G.E., Coelli T.J., 1988. Prediction of firm-level technical efficiencies with a generalized frontier production function and panel data. J. Econom. 38, 387–399. 10.1016/0304-4076(88)90053-X.

[65] Nielsen H.B., Mortensen S.B., Mortensen M.S.B., 2016. Package ‘ucminf.’.

[66] Hensher D.A., Rose J.M., Greene W.H., 2015. Applied choice analysis, 2nd editio. ed. Cambridge University Press. 10.1017/CBO9781316136232.

[67] Hagenaars J. A., & McCutcheon A. L. (Eds.), 2002. Applied latent class analysis. Cambridge University Press. New York, NY.

[68] Mercadé L., Delgado M., Gil J.M., 2012. Practiques de fertilització a Catalunya. Enquesta 2010. Generalitat de Catalunya: Departament d’Agricultura, Alimentació i Acció Rural.

[69] Spanish Ministry of Agriculture, 2010. Balance del nitrógeno en la agricultura española (año 2008)—criterios utilizados.

[70] Cummings R. G., & Taylor L. O., 1999. Unbiased value estimates for environmental goods: a cheap talk design for the contingent valuation method. Am. Econ. Rev, 89(3), 649–665.

[71] Lusk J. L., 2003. Effects of cheap talk on consumer willingness‐to‐pay for golden rice. Am. J. Agric. Econ, 85(4), 840–856.

[72] Michalos A.C., 2014. Encyclopedia of quality of life research. Dordrecht: Springer Netherlands.

[73] Bogetoft P., Hougaard J.L., 2003. Rational Inefficiencies. J. Product. Anal. 2003 203 20, 243–271. 10.1023/A:1027347616038.

[74] Hansson H., Manevska-Tasevska G., Asmild M., 2020. Rationalising inefficiency in agricultural production—The case of Swedish dairy agriculture. Eur. Rev. Agric. Econ. 47, 1–24. 10.1093/erae/jby042.

[75] Cuesta R.A., 2000. A Production Model With Firm-Specific Temporal Variation in Technical Inefficiency: With Application to Spanish Dairy Farms. J. Product. Anal. 13, 139–158. 10.1023/A:1017297831646.

[76] Mathijs E., Swinnen J.F.M., 2001. Production Organization and Efficiency During Transition: An Empirical Analysis of East German Agriculture. Rev. Econ. Stat. 83, 100–107. 10.1162/003465301750160072.

[77] Roibas D., Alvarez A., 2010. Impact of genetic progress on the profits of dairy farmers. J. Dairy Sci. 93, 4366–4373. doi: 10.3168/jds.2010-3135 20723710

[78] Florax R. J., Travisi C. M., & Nijkamp P., 2005. A meta-analysis of the willingness to pay for reductions in pesticide risk exposure. Eur. Rev. Agric. Econ, 32(4), 441–467.

[79] Burton R.J.F., 2014. The influence of farmer demographic characteristics on environmental behaviour: A review. J. Environ. Manage. 135, 19–26. doi: 10.1016/j.jenvman.2013.12.005 24508843

[80] Lansink A.O., Pietola K., Bäckman S., 2002. Efficiency and productivity of conventional and organic farms in Finland 1994–1997. Eur. Rev. Agric. Econ. 10.1093/erae/29.1.51.

[81] Mayen C.D., Balagtas J. V., Alexander C.E., 2010. Technology Adoption and Technical Efficiency: Organic and Conventional Dairy Farms in the United States. Am. J. Agric. Econ. 92, 181–195. 10.1093/ajae/aap018.

[82] Serra T., Goodwin B.K., 2009. The efficiency of Spanish arable crop organic farms, a local maximum likelihood approach. J. Product. Anal. 31, 113–124. 10.1007/s11123-008-0124-4.

[83] Van Cauwenbergh N., Biala K., Bielders C., Brouckaert V., Franchois L., Garcia Cidad V., et al. SAFE-A hierarchical framework for assessing the sustainability of agricultural systems. Agric. Ecosyst. Environ. 120, 229–242. 10.1016/j.agee.2006.09.006.

[84] Govindan K., Shankar M., Kannan D., 2018. Supplier selection based on corporate social responsibility practices. Int. J. Prod. Econ. 200, 353–379. 10.1016/j.ijpe.2016.09.003.

